# Screening for SARS-CoV-2 by RT-PCR: Saliva or nasopharyngeal swab? Rapid review and meta-analysis

**DOI:** 10.1371/journal.pone.0253007

**Published:** 2021-06-10

**Authors:** Nusaïbah Ibrahimi, Agnès Delaunay-Moisan, Catherine Hill, Gwénaël Le Teuff, Jean-François Rupprecht, Jean-Yves Thuret, Dan Chaltiel, Marie-Claude Potier

**Affiliations:** 1 Service de Biostatistique et d’Épidémiologie, Institut Gustave Roussy, Université Paris-Saclay, Villejuif, France; 2 Université Paris-Saclay, CEA, CNRS, Institute for Integrative Biology of the Cell (I2BC), Gif-sur-Yvette Cedex, France; 3 Aix Marseille Univ, Université de Toulon, CNRS, Centre de Physique Théorique, Turing Center for Living Systems, Marseille, France; 4 Institut du Cerveau (ICM), CNRS UMR 7225 – Inserm U1127, Sorbonne Université, Paris, France; "INSERM", FRANCE

## Abstract

**Background:**

Diagnosis of COVID-19 in symptomatic patients and screening of populations for SARS-CoV-2 infection require access to straightforward, low-cost and high-throughput testing. The recommended nasopharyngeal swab tests are limited by the need of trained professionals and specific consumables and this procedure is poorly accepted as a screening method In contrast, saliva sampling can be self-administered.

**Methods:**

In order to compare saliva and nasopharyngeal/oropharyngeal samples for the detection of SARS-CoV-2, we designed a meta-analysis searching in PubMed up to December 29th, 2020 with the key words “(SARS-CoV-2 OR COVID-19 OR COVID19) AND (salivary OR saliva OR oral fluid)) NOT (review[Publication Type]) NOT (PrePrint[Publication Type])” applying the following criteria: records published in peer reviewed scientific journals, in English, with at least 15 nasopharyngeal/orapharyngeal swabs and saliva paired samples tested by RT-PCR, studies with available raw data including numbers of positive and negative tests with the two sampling methods. For all studies, concordance and sensitivity were calculated and then pooled in a random-effects model.

**Findings:**

A total of 377 studies were retrieved, of which 50 were eligible, reporting on 16,473 pairs of nasopharyngeal/oropharyngeal and saliva samples. Meta-analysis showed high concordance, 92.5% (95%CI: 89.5–94.7), across studies and pooled sensitivities of 86.5% (95%CI: 83.4–89.1) and 92.0% (95%CI: 89.1–94.2) from saliva and nasopharyngeal/oropharyngeal swabs respectively. Heterogeneity across studies was 72.0% for saliva and 85.0% for nasopharyngeal/oropharyngeal swabs.

**Interpretation:**

Our meta-analysis strongly suggests that saliva could be used for frequent testing of COVID-19 patients and “en masse” screening of populations.

## Introduction

Propagation of infections by SARS-CoV-2, the coronavirus causing the COVID-19 pandemic, occurs from asymptomatic as well as symptomatic carriers [1]. To reduce the circulation of the virus in the population, SARS-CoV-2 carriers need to be identified rapidly and isolated as soon as possible, ideally before the onset of symptoms. When the virus has disseminated throughout a whole country, massive testing becomes of utmost urgent importance to combat the pandemic [2–4].

The recommended diagnosis of SARS-CoV-2 infection from the World Health Organisation is based on real time RT-qPCR detection of viral RNA in respiratory specimen such as nasopharyngeal swabs (NP), bronchial aspiration (BA), throat swab and sputum [5]. The American Centres For Disease Control and Prevention and the European Centre for Disease Prevention and Control now recommend viral testing from the respiratory system such as nasal or oral swabs or saliva [6, 7]. The French health regulatory authority (*Haute Autorité de Santé*) has recently included in its recommendations the use of saliva samples for the detection of SARS-CoV-2 in symptomatic individuals for whom nasopharyngeal sampling is difficult, and for mass testing within schools, universities and among health workers [8].

To make the diagnostic acceptable to the largest number of people, especially asymptomatic individuals, massive testing should be based on a sampling procedure that is inexpensive, easy to set up and well accepted by the population [9]. In contrast to nasopharyngeal swabbing, saliva sampling meets these criteria. Saliva sampling is fast, non-invasive, inexpensive and painless. It does not require trained professional with personal protective equipment nor other material than a simple plastic tube, and can be self-administered.

To evaluate saliva sampling for the detection of SARS-CoV-2, we conducted a meta-analysis on studies published in peer-reviewed journals until the 29^th^ of December 2020 comparing the detection of SARS-CoV-2 using RT-PCR on paired nasopharyngeal/oropharyngeal and saliva samples in the same individuals sampled at the same time.

## Methods

### Search strategy and selection criteria

Literature search in PubMed (https://pubmed-ncbi-nlm-nih-gov) run the 29^th^ of December 2020 with search word (SARS-CoV-2 OR COVID-19 OR COVID19) AND (salivary OR saliva OR oral fluid)) NOT (review[Publication Type]) NOT (PrePrint[Publication Type]) identified 377 articles. We included publications if they met the following eligibility criteria:

1. Records published in peer reviewed scientific journals;
2. Records published in English;
3. Data curation based on examination of the title and abstract, searching for research articles assessing viral RNA presence in saliva vs nasopharyngeal and/or oropharyngeal swabs;
4. Availability of nasopharyngeal (and/or oropharyngeal) swabs and saliva data on specimens sampled on the same individuals at the same time;
5. Detection of SARS-CoV-2 using the same RT-PCR method on both samples;
6. More than 15 individuals included in the study.

### Data analysis

From each eligible article, we extracted: the number of individuals positive for SARS-CoV-2 in both nasopharyngeal/oropharyngeal swabs and saliva (a), those positive only in nasopharyngeal/oropharyngeal swab (b), those positive only in saliva (c) and (d) those negative in both nasopharyngeal swab and saliva (Table 1). From these data, we calculated the concordance of the same test (RTqPCR for 49 studies and RTdPCR for 1 study) on the two types of sample (a+d)/(a+b+c+d). We also computed the sensitivity of the test on each type of sample. The estimation of the sensitivity of a test requires a reference diagnosis. Since nasopharyngeal swab sampling has been shown to produce false negatives by RTqPCR [10], sensitivities for the saliva and the nasopharyngeal swab are defined here respectively as (a+c)/(a+b+c) and (a+b)/(a+b+c), considering as true positive any individual with a positive result on one or the other sample. This definition of a positive individual is also in agreement with the US-CDC and the ECDC directives on SARS-Cov-2 testing.

**Table 1:** Studies comparing SARS-CoV-2 detection in paired saliva and nasopharyngeal samples meeting inclusion criteria.

Reference	Number of tested individuals					Concordance	Reference: S+ ou N+	
	Saliva + Nasoph. +	Saliva—Nasoph. +	Saliva + Nasoph. -	Saliva—Nasoph. -	Total		Sensitivity of S	Sensitivity of N
	a	b	c	d	n = a+b+c+d	(a+d)/n	(a+c)/p	(a+b)/p
Aita	7	1	0	35	43	97.7%	87.5%	100.0%
Altawalah	287	57	18	529	891	91.6%	84.3%	95.0%
Azzi	22	4	33	54	113	67.3%	93.2%	44.1%
Babady	16	1	1	69	87	97.7%	94.4%	94.4%
Barat	30	7	1	421	459	98.3%	81.6%	97.4%
Berenger	52	11	6	6	75	77.3%	84.1%	91.3%
Bhattacharya	53	5	0	16	74	93.2%	91.4%	100.0%
Binder	10	1	1	7	19	89.5%	91.7%	91.7%
Borghi	79	28	7	187	301	88.4%	75.4%	93.9%
Braz-Silva	37	15	18	131	201	83,6%	78,6%	74,3%
Byrne	12	2	0	96	110	98.2%	85.7%	100.0%
Cassinarri	8	5	1	17	31	80.6%	64.3%	92.9%
Caulley	34	22	14	1869	1939	98.1%	68.6%	80.0%
Chau	19	0	1	7	27	96.3%	100.0%	95.0%
Chen	49	6	3	0	58	84.5%	89.7%	94.8%
Güçlü	23	4	4	33	64	87.5%	87.1%	87.1%
Hanege	29	9	0	0	38	76.3%	76.3%	100.0%
Hanson	75	5	6	268	354	96.9%	94.2%	93.0%
Hasanoglu	27	21	3	9	60	60.0%	58.8%	94.1%
Iwasaki	8	1	1	66	76	97.4%	90.0%	90.0%
Jamal 1	44	20	8	19	91	69.2%	72.2%	88.9%
Jamal 2	42	14	9	10	75	69.3%	78.5%	86.2%
Kandel	39	4	3	383	429	98.4%	91.3%	93.5%
Kojima	20	3	6	16	45	80.0%	89.7%	79.3%
Landry	28	5	2	89	124	94.4%	85.7%	94.3%
Leung	38	7	13	37	95	78.9%	87.9%	77.6%
Matic	15	6	1	52	74	90.5%	72.7%	95.5%
McCormick-Baw	47	2	1	106	156	98.1%	96.0%	98.0%
Migueres	34	7	3	79	123	91.9%	84.1%	93.2%
Moreno-Contreras	19	9	6	37	71	78.9%	73.5%	82.4%
Nagura-Ikeda	84	19	0	0	103	81.6%	81.6%	100.0%
Otto	45	0	4	43	92	95.7%	100.0%	91.8%
Pasomsub	16	3	2	179	200	97.5%	85.7%	90.5%
Procop	38	0	1	177	216	99.5%	100.0%	97.4%
Rao	73	11	76	57	217	59.9%	93.1%	52.5%
Sakanashi	15	0	4	9	28	85.7%	100.0%	78.9%
Senok	19	7	9	366	401	96.0%	80.0%	74.3%
Skolimowska	15	3	1	112	131	96.9%	84.2%	94.7%
Sorelle	32	7	0	44	83	91.6%	82.1%	100.0%
Sui	14	0	2	0	16	87.5%	100.0%	87.5%
Torres	46	54	8	835	943	93.4%	50.0%	92.6%
Uwamino	32	15	11	138	196	86.7%	74.1%	81.0%
Vaz	67	4	2	82	155	96.1%	94.5%	97.3%
Vogels	49	5	4	3776	3834	99.8%	91.4%	93.1%
Williams	33	6	1	49	89	92.1%	85.0%	97.5%
Wong	104	18	37	70	229	76.0%	88.7%	76.7%
Wyllie	34	9	13	13	69	68.1%	83.9%	76.8%
Yee	69	18	10	203	300	90.7%	81.4%	89.7%
Yokota	42	4	6	1872	1924	99.5%	92.3%	88.5%
Zhu	382	60	15	487	944	92.1%	86.9%	96.7%
Total	2412	525	376	13160	16473	94,5%	84,2%	88,7%

The overall concordance and sensitivities have been estimated in a meta-analysis via Generalized Linear Mixed Models (GLMM), using a fixed-effect model, and also a random-effect model in case of over dispersion of the observations [11, 12]. Dispersion of effect sizes was evaluated using the Higgins *I*^*2*^ estimate of heterogeneity along with the Cochran’s Q in fixed-effect models [13] and using the tau^2^ estimate (between-study variance which is the variance of the distribution of true effect size) in random-effects models. The tau^2^ was calculated using the maximum likelihood estimator. As some studies have a sample size too small for the normality hypothesis, confidence intervals for each study were computed using the Clopper-Pearson method [14] also called “exact” binomial interval. Those for the overall estimates are based on normal approximation. Results are presented as forest plots.

All analyses were done with R version 4.0.3, using the package “meta” version 4.15–1 (2020-09-30) [15, 16].

## Results

**Fig 1:**
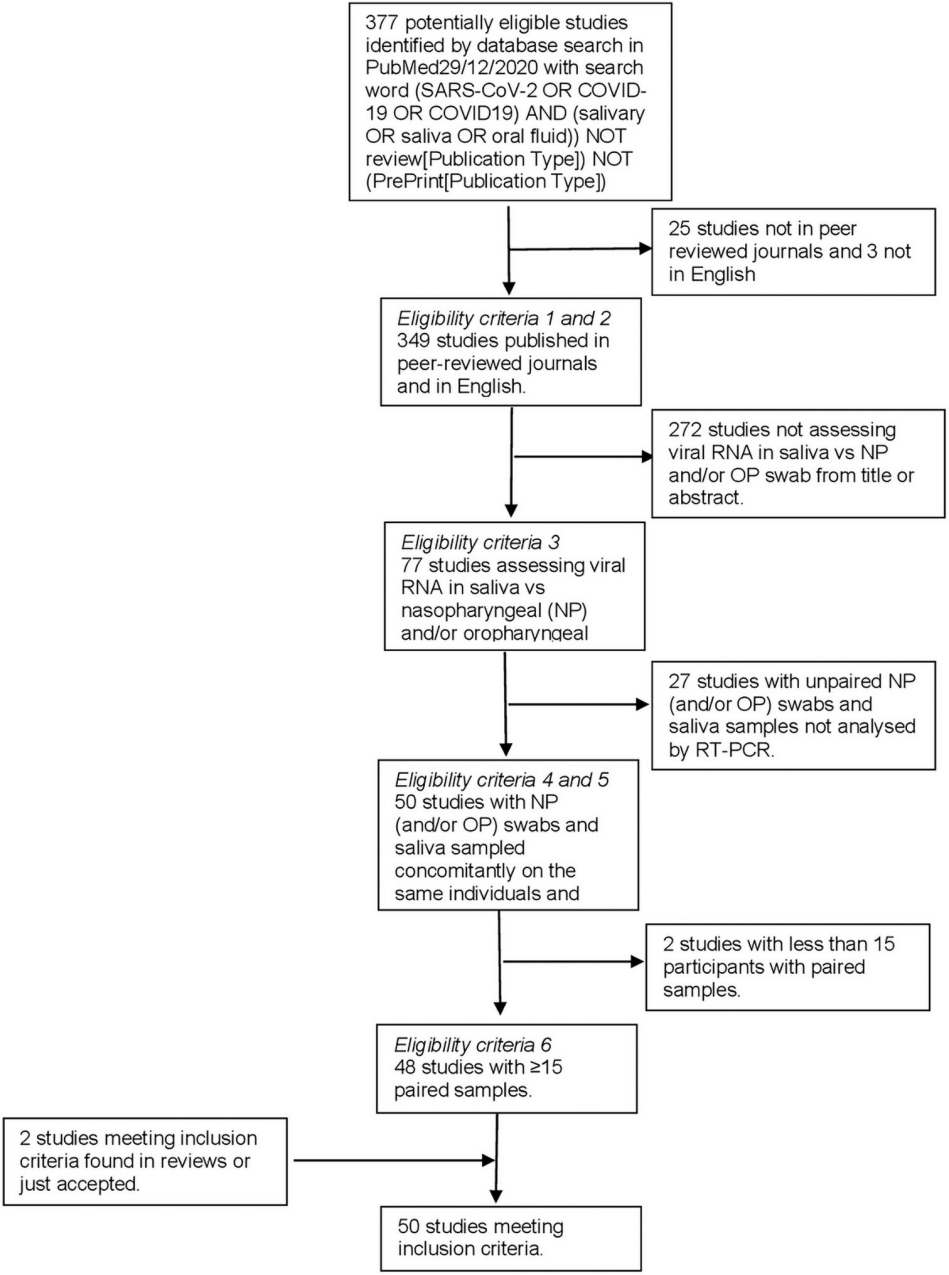
Evidence search and selection.

**Fig 2:**
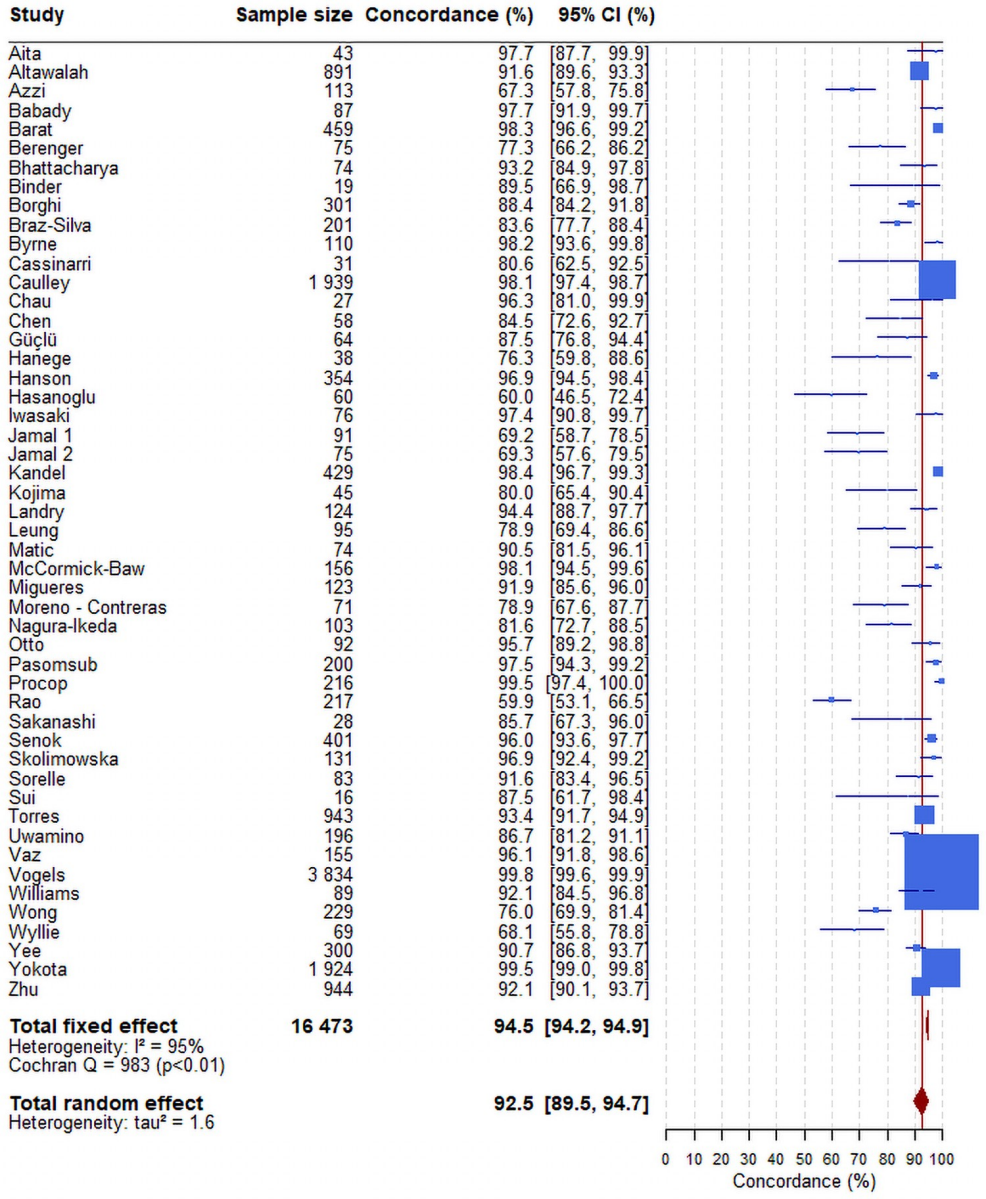
Forest plot of the concordance between the results of RTqPCR tests on nasopharyngeal and saliva samples. The confidence intervals for each study are computed using the Clopper-Pearson method. Those for the overall estimates (fixed-effect or random-effect) are based on normal approximation. The blue box size is proportional to the number of positive tests. The red line corresponds to the value of the overall concordance of the random-effect model. This vertical line enables to locate the studies having an estimate concordance higher than 92.5%.

Forty-eight studies comparing SARS-CoV-2 loads in NP swabs and saliva samples collected concurrently in the same individuals using the same technique and providing positivity and negativity in both samples have been identified in PubMed [17–64]. We also included 2 articles not identified in the original Pubmed keywords search while fulfilling eligibility criteria (Table 1 and Fig 1) [65, 66]. In total 16,473 paired samples were analysed. The number of paired samples per study varied between 16 and 3834. Meta-analysis showed an overall concordance of 92.5% (95%CI: 89.5–94.7) across studies (Fig 2).

**Fig 3:**
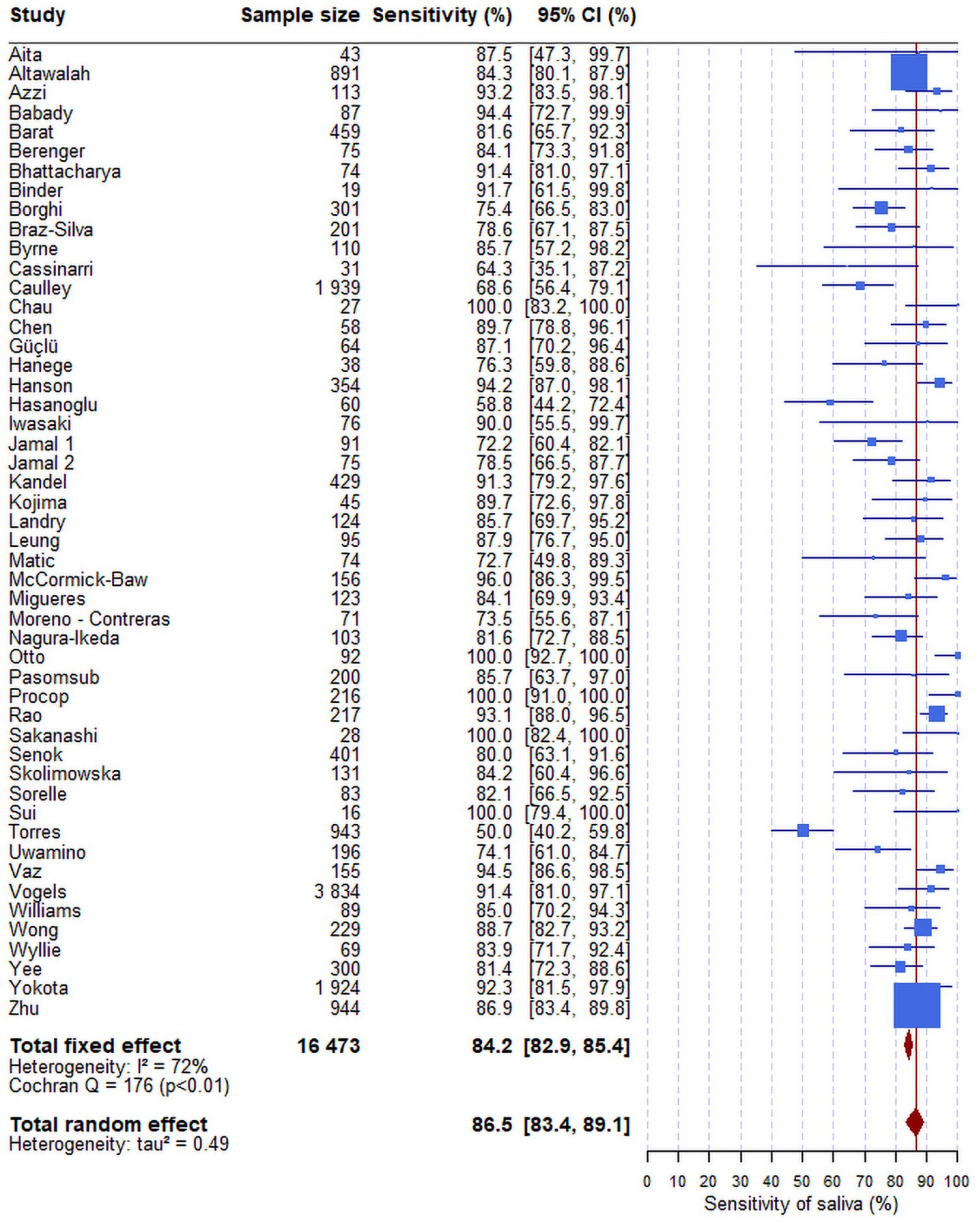
Forest plot of the sensitivity of RTqPCR test on saliva. The confidence intervals for each study are computed using the Clopper-Pearson method. Those for the overall estimates (fixed-effect or random-effect) are based on normal approximation. The blue box size is proportional to the number of positive tests. The difference between fixed-effect and random-effect overall sensitivity (respectively 84.2%, 86.5%) is low. The red line corresponds to the value of the overall sensitivity of the random-effect model. This vertical line enables to locate the studies having an estimate sensitivity higher than 86.5%. The heterogeneity estimator I^2^ is equal to 72%, which means a higher level of heterogeneity.

**Fig 4:**
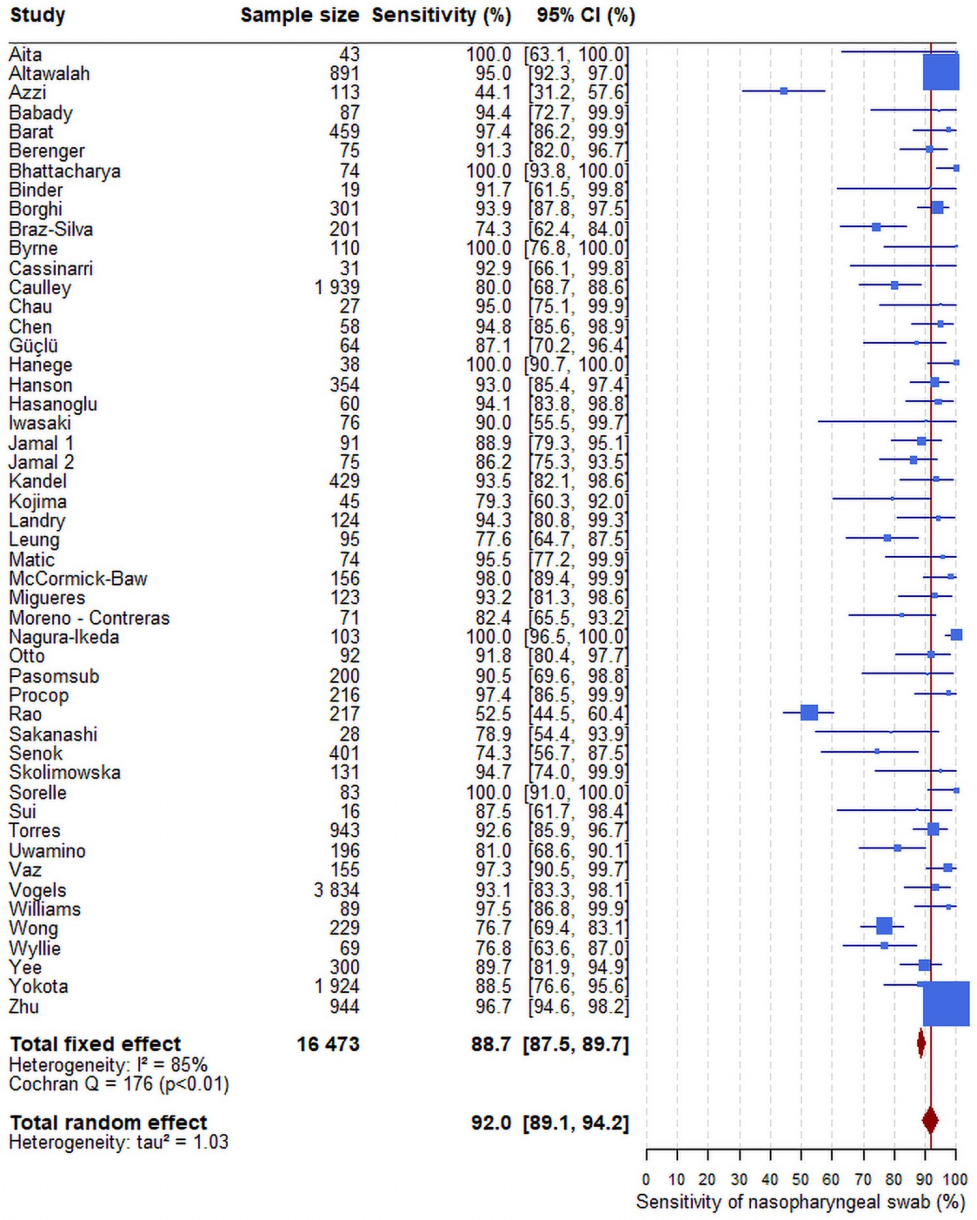
Forest plot of the sensitivity of RTqPCR test on nasopharyngeal sample. The confidence intervals for each study are computed using the Clopper-Pearson method. Those for the overall estimates (fixed-effect or random-effect) are based on the normal approximation. The blue box size is proportional to the number of positive tests. The red line corresponds to the value of the overall sensitivity of the random-effect model. This vertical line enables to locate the studies having an estimate sensitivity higher than 92.0%. The heterogeneity estimator I^2^ is equal to 85%, which means a higher level of heterogeneity.

The overall sensitivity of the RT-PCR test from saliva samples was 86.5% (95%CI: 83.4–89.1) (Fig 3) versus 92.0% from nasopharyngeal swabs (95%CI: 89.1–94.2) (Fig 4). There was no association between the sensitivities of the saliva (S1 Fig) or of nasopharyngeal swab (S2 Fig) estimated in each study and the prevalence of the virus in the same study. If the sensitivity of the saliva was lower in populations of asymptomatic individuals than in population of individuals with symptoms, one would expect to observe a lower sensibility of the saliva in the studies with a low prevalence of infection.

In our fixed-effect model meta-analysis, saliva gave I^2^ of 72% and nasopharyngeal gave I^2^ of 85%, both corresponding to high heterogeneity. Therefore, a random-effect model was performed to assess the overall sensitivities of the RTqPCR tests, taking into account the fact that the studies did not originate from one single population. The variance of saliva was 0.49 and that of nasopharyngeal was 1.03. As a sensitivity analysis, we also used various other methods to estimate the sensitivity, the confidence interval and the tau^2^ and all yielded very similar results (Figs 2–4).

## Discussion

This meta-analysis reviewed 50 studies and concluded to a high concordance between nasopharyngeal and saliva samples for the detection of SARS-CoV-2 by RT-PCR. Although sensitivity was slightly lower on saliva samples than on nasopharyngeal samples, both values are above the 80% sensitivity cut-off recommended by health regulatory authorities such as the French Haute Autorité de Santé [67].

For computing concordance and sensitivities, we considered here the reference as SARS-CoV-2 positivity by RT-PCR either in saliva and/or nasopharyngeal samples since the presence of the virus in any sample is indicative of virus carriage.

In the context of mass screening, most participants are asymptomatic. Among the 50 studies analysed, only one included exclusively asymptomatic participants [63] and 8 studies included both symptomatic and asymptomatic participants but it was impossible to separate the data between the two populations [17, 20, 25, 29, 51, 56, 58, 65]. One study of contact cases included a larger number of asymptomatic subjects as compared to study of symptomatic subjects [49]. We did not observe any difference in concordance of the tests in these particular studies involving asymptomatic participants (Table 1). Formal comparison of nasopharyngeal and saliva samples from asymptomatic individuals is challenging: it would require to screen a large population for a small number of positive cases detected since the prevalence is usually low in this population. On the contrary, the symptomatic population expectedly contains higher percentage of positive subjects, as symptoms usually timely correlates with the highest viral load, which allow an easier comparison of both sampling procedures. Anyhow, viral load in saliva of presymptomatic subjects remains in the range of detection of the RT-PCR test for several days both in saliva [68] and nasopharyngeal samples [69]. In addition, both asymptomatic and symptomatic subjects appear to be contagious [1] with similarities in their viral load evolution [70–72]. Moreover, S3 Fig shows that in France the proportion of positive cases in the symptomatic tested population is consistently about 5 times larger than in the asymptomatic tested population, independently of viral prevalence over time. This and the fact that neither saliva nor nasopharyngeal sensitivities are affected in a screening-like context (the low prevalence being taken as a proxy, S1 and S2 Figs) strongly predict that viral detection is expected to exhibit similar performance in both populations.

Our meta-analysis showed large heterogeneity between studies. Sources of heterogeneity are both biological and technical. Biological heterogeneity may come from the fact that a given individual may carry the virus in only one of the saliva or nasopharyngeal specimens, or from the timing of sampling during the course of contamination. Technical heterogeneity comes from differences in the sampling and in RT-PCR methods. Among the 50 studies meeting the inclusion criteria for the meta-analysis, 29 studies report saliva collection in sterile containers (urine tubes or vials) without any additional solution. The other studies diluted the saliva in various viral transport media or phosphate buffer saline with or without bovine serum albumin. Other sources of variability come from differences in the amplified region of SARS-Cov-2 or to different positivity threshold between studies; most studies do not present viral load data but only cycle thresholds (Ct) for specific amplification of SARS-Cov-2 sequences and not even Ct differences (ΔCt) with a human reference gene.

Altogether, our meta-analysis of 50 studies including 16,473 paired samples shows high concordance (92.5%) between nasopharyngeal/oropharyngeal swabs and saliva, with a 5% higher sensitivity for the nasopharyngeal/oropharyngeal (92.0%) as compared to saliva (86.5%). While that might have been a liability in the context of individual diagnosis, it is not such a concern for mass screening, especially given the major advantage of the saliva sampling in terms of logistics. Previous meta-analyses of paired nasopharyngeal and saliva samples included less than 15 peer-reviewed studies or preprints and reported average sensitivity of 91%, 85% and 83.4% in 4, 16 and 5 studies respectively [73–75]. However, these studies used nasopharyngeal positivity as the reference, which did not seem relevant in our context.

To prevent a shortage of analytic reagents and to cut the costs necessarily associated to mass screening strategies, several recent publications have proposed mass testing methods based on saliva sampling either through extraction-free protocols [76–78] or through pre-extraction sample pooling [79–81]. In addition, sample pooling has gained a recognized interest for recurrent screening programs from the Centres of Disease Control recommendations [82], and surveillance protocols implemented in higher education institutions across the world, e.g. the State University of New York (United States) [83], Liège University (Belgium) [84], Heidelberg University (Germany) [85] as well as at Nottingham University (United Kingdom) [86].

In conclusion, this meta-analysis conclusively demonstrates that saliva is as valid as nasopharyngeal sampling for the detection of SARS-CoV-2 infections in symptomatic as well as asymptomatic carriers. In contrast to nasopharyngeal swabs, saliva sampling is simple, fast, non-invasive, inexpensive, painless and it thus uniquely applicable for surveillance, screening and diagnosis.

## Supporting information

S1 Fig Saliva sensitivity in each of the 50 studies as a function of SARS-CoV2 prevalence. (TIF)

S2 Fig Nasopharyngeal sample sensitivity in each of the 50 studies as a function of SARS-CoV2 prevalence. (TIF)

S3 Fig Prevalence in the population tested in France by symptomatic status. Week 28 corresponds to the results published the 13^th^ of July 2020. Sources: Points épidémiologiques hebdomadaires.
(TIF)

S1 Checklist PRISMA 2009 checklist. (DOC)
