# Supplementary figures and images for: Screening for SARS-CoV-2 by RT-PCR: Saliva or nasopharyngeal swab? Rapid review and meta-analysis
Source: PLoS One. 2021 Jun 10;16(6):e0253007. doi: 10.1371/journal.pone.0253007 (PMC8191978; doi:10.1371/journal.pone.0253007)

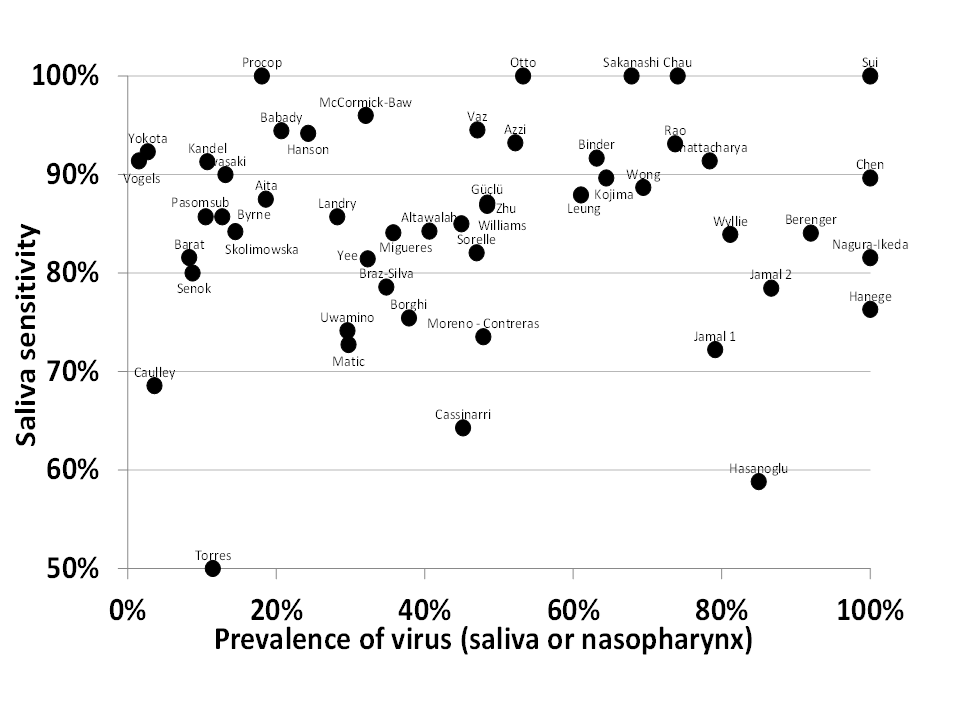

Supplement: S1 Fig — (TIF) [file pone.0253007.s001.tif]

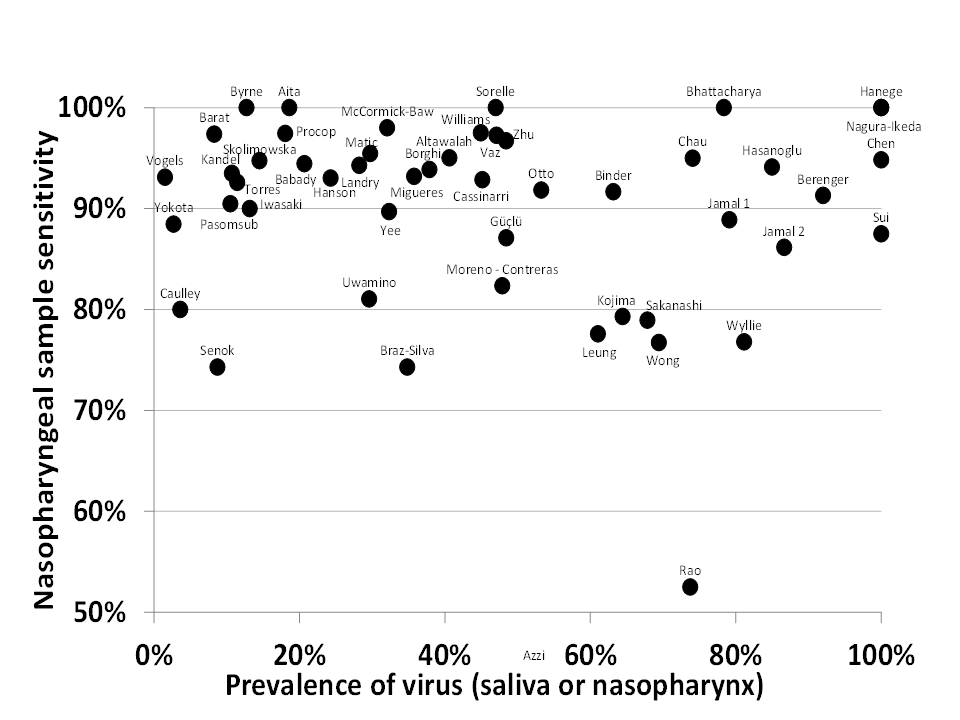

Supplement: S2 Fig — (TIF) [file pone.0253007.s002.tif]

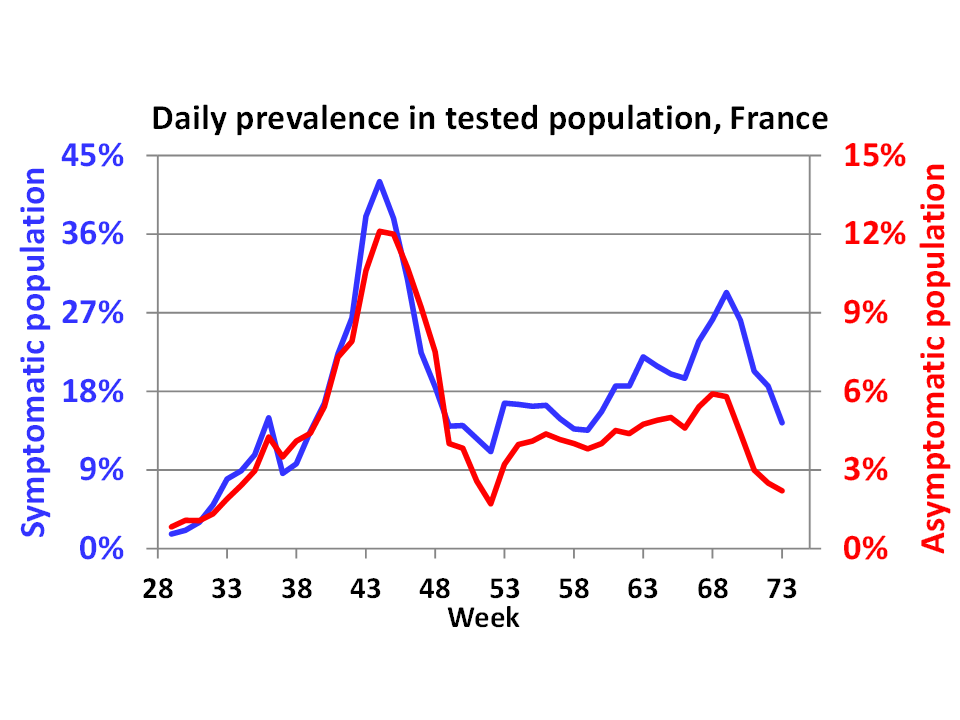

Supplement: S3 Fig — Week 28 corresponds to the results published the 13th of July 2020. Sources: Points épidémiologiques hebdomadaires. (TIF) [file pone.0253007.s003.tif]
